# Supplementary material for: Obstructive sleep apnea improves with non-invasive hypoglossal nerve stimulation using temporal interference
Source: Bioelectron Med. 2023 Aug 9;9:18. doi: 10.1186/s42234-023-00120-7 (PMC10410873; doi:10.1186/s42234-023-00120-7)
Supplement: Supplementary file 2 [file 42234_2023_120_MOESM2_ESM.docx]

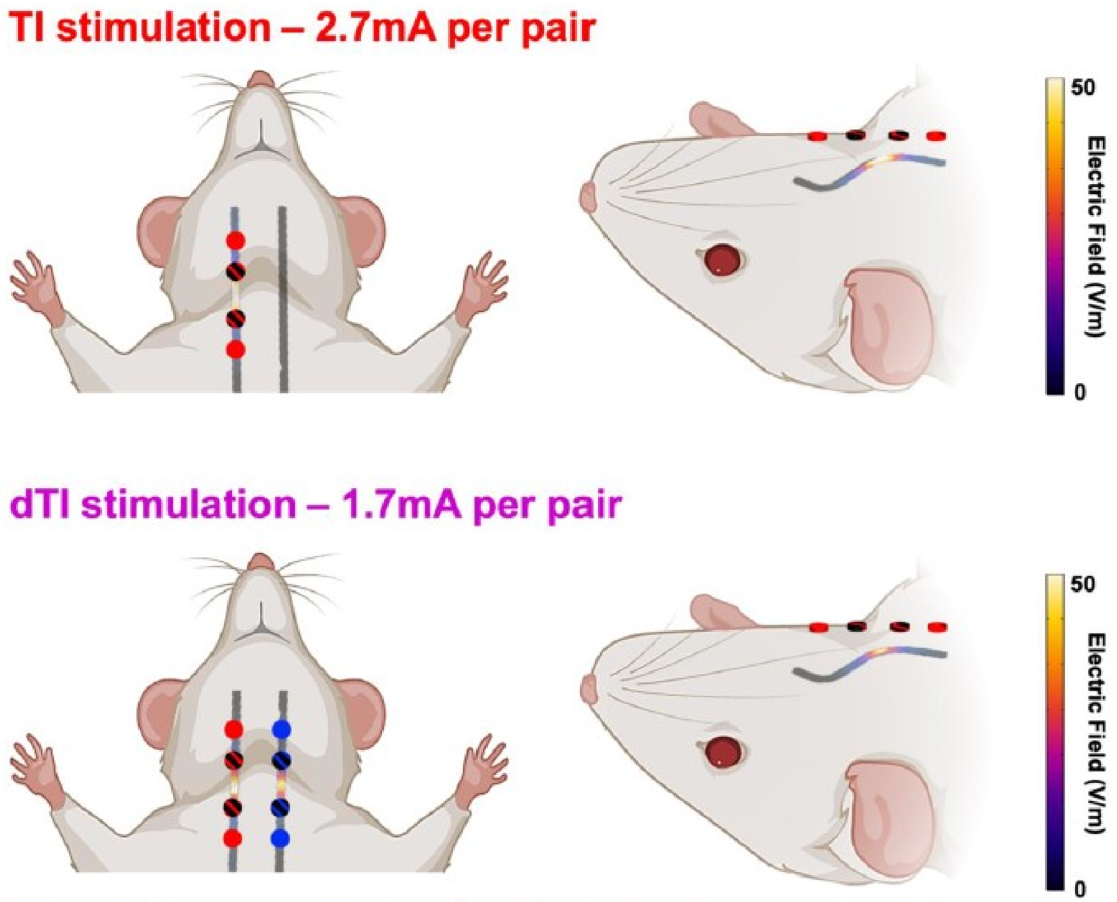

Figure S2. Finite element simulation comparison of bTI unilateral TI exposures.
