# Supplementary material for: Obstructive sleep apnea improves with non-invasive hypoglossal nerve stimulation using temporal interference
Source: Bioelectron Med. 2023 Aug 9;9:18. doi: 10.1186/s42234-023-00120-7 (PMC10410873; doi:10.1186/s42234-023-00120-7)
Supplement: Supplementary file 3 [file 42234_2023_120_MOESM3_ESM.docx]

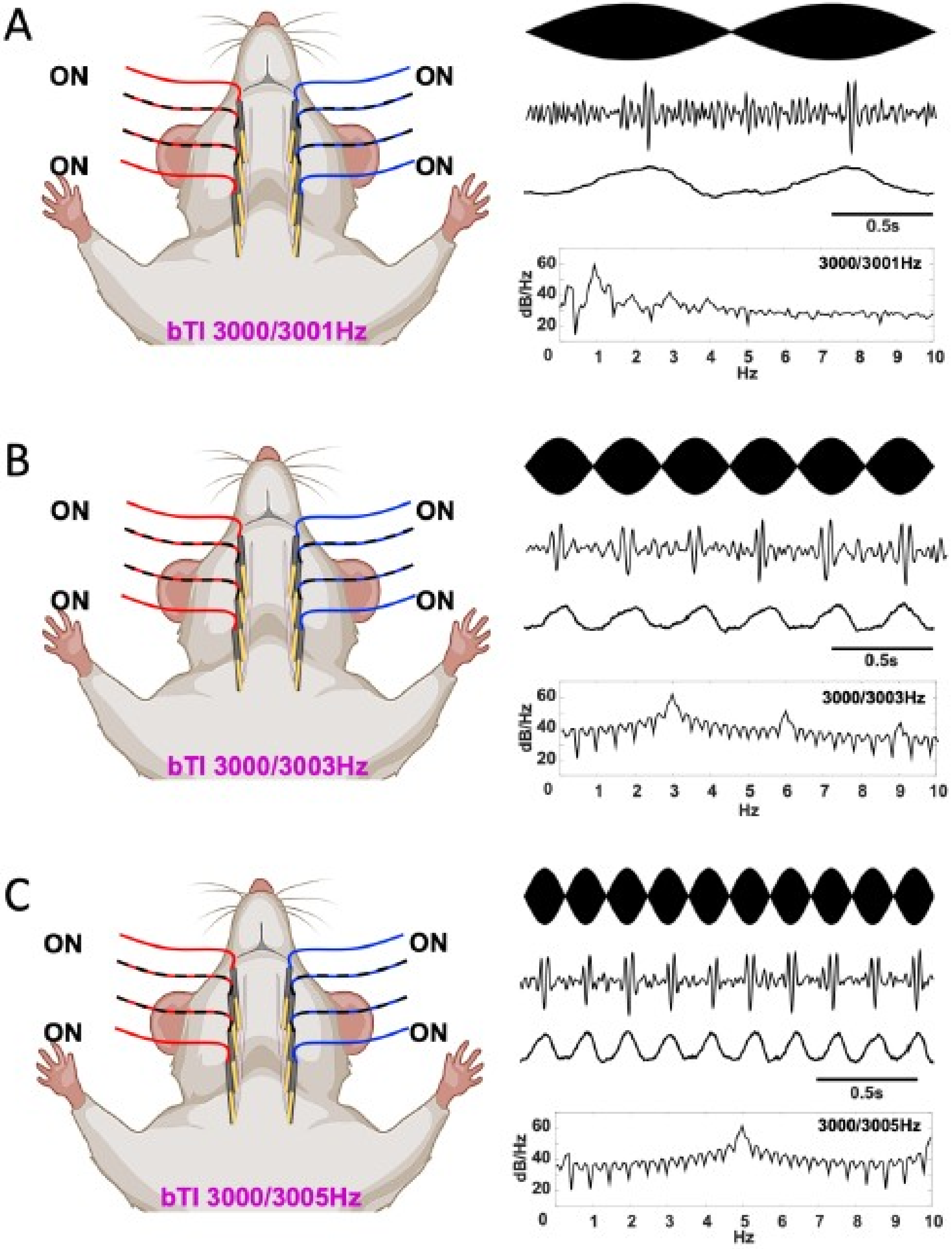

Figure S3. Different offset frequencies induce tongue movement. When changing the offset frequency (*Δf*), the evoked tongue movements and muscle activity changes frequency. Looking at very low offset frequency, namely 1Hz, the stimulation is too low to only evoke one muscle activity, inducing rather a continuous stimulation with several CMAPs than a rhythmic one at the offset frequency. The depicted EMG and FR are
